# Antimicrobial Resistant *Staphylococcus* Species Colonization in Dogs, Their Owners, and Veterinary Staff of the Veterinary Teaching Hospital of Naples, Italy

**DOI:** 10.3390/pathogens12081016

**Published:** 2023-08-05

**Authors:** Francesca Paola Nocera, Francesca Pizzano, Angelo Masullo, Laura Cortese, Luisa De Martino

**Affiliations:** 1 Department of Veterinary Medicine and Animal Production, University of Naples ‘Federico II’, Via F. Delpino 1, 80137 Naples, Italy; 2 Task Force on Microbiome Studies, University of Naples ‘Federico II’, 80137 Naples, Italy

**Keywords:** *Staphylococcus* spp., transmission, antimicrobial resistance, dogs, pet owners, veterinary staff

## Abstract

This study aimed to identify *Staphylococcus* species isolated from nasal swabs of both healthy and diseased dogs, and those of human origin, obtained from nasal swabs of both owners and veterinary staff. Firstly, pet owners were requested to complete a questionnaire relating to the care and relationship with their pets, whose results mainly showed a statistically significant higher frequency of hand washing in diseased dogs’ owners than in healthy dogs’ owners. Canine nasal swabs were obtained from 43 diseased dogs and 28 healthy dogs, while human nasal swabs were collected from the respective dogs’ owners (71 samples) and veterinary staff (34 samples). The isolation and identification of *Staphylococcus* spp. were followed by disk diffusion method to define the antimicrobial resistance profiles against 18 different molecules. *Staphylococcus pseudintermedius* was the most frequent isolated strain in both diseased (33.3%) and healthy (46.1%) dogs. *Staphylococcus epidermidis* was the most frequent isolated bacterium in diseased dogs’ owners (66.6%), while in nasal samples of healthy dogs’ owners, the same frequency of isolation (38.4%) was observed for both *Staphylococcus epidermidis* and *Staphylococcus aureus*. All the isolated strains showed good susceptibility levels to the tested antimicrobials; however, the carriage of oxacillin-resistant strains was significantly higher in diseased dogs than in healthy ones (71% and 7.7%, respectively). Only in three cases the presence of the same bacterial species with similar antimicrobial resistance profiles in dogs and their owners was detected, suggesting the potential bacterial transmission. In conclusion, this study suggests potential transmission risk of staphylococci from dogs to humans or *vice versa*, and highlights that the clinical relevance of *Staphylococcus pseudintermedius* transmission from dog to human should not be underestimated, as well as the role of *Staphylococcus aureus* from human to dog transmission.

## 1. Introduction

Staphylococci are normal inhabitants of skin and mucus membranes of both humans and animals [1]. In dogs, nasal carriage of both coagulase-positive and coagulase-negative staphylococci can be influenced not only by diseases but also by some environmental factors, such as the close contact with humans or *vice versa* [2]. Although it is still not clear how the transmission of staphylococci between dogs and humans occurs, previous studies suggest that close contact might cause a higher colonization of *Staphylococcus aureus* in dogs and *Staphylococcus pseudintermedius* in humans [2,3,4].

*Staphylococcus aureus* (*S. aureus*) is widely disseminated as a colonizer and as an opportunistic pathogen among humans and animals [3]. In the human community, it colonizes the anterior nares in 20% to 80% of healthy humans [5], and these people can be defined as “carriers”, as in normal physiological conditions their immunity system is able to fight off this opportunistic pathogen. However, in specific conditions (e.g., injuries, skin disorders, metabolic diseases), this microorganism, finding propitious conditions for its reproduction, can give rise to both mild and life-threatening infections. Currently, the spread of methicillin-resistant *S. aureus* (MRSA), showing also multidrug-resistant profile, has increased worldwide and, in particular, it has emerged not only as a nosocomial pathogen as healthcare-associated MRSA, but also as community-associated MRSA and livestock-acquired MRSA [6,7].

Rates of methicillin resistance among clinical *S. aureus* isolates vary greatly among countries, from rates as low as 9% in Scandinavian countries [8] to over 50% in countries such as the United States and China [9,10]. Although nosocomial MRSA infections are declining in the United States, Europe, China, and many other countries, possibly due to increased surveillance and hygiene measures, they are still increasing in less-developed countries [11].

Human-to-human transmission of MRSA occurs through direct contact with carriers, infected people, or with contaminated medical devices and equipment. Therefore, MRSA is of greatest concern in hospital settings, where patients with weakened immune systems are more prone to infection than the healthy population. Furthermore, animals, mainly food-producing ones, have been described as MRSA reservoirs [12]. The close contact with an MRSA-positive animal can favor colonization by this pathogen in humans, but they rarely become infected [13]. Therefore, farmers and livestock breeders, but also veterinary staff, are exposed to a higher risk of being colonized by MRSA. Indeed, Neradova et al. [14] reported that the prevalence of MRSA nasal carriage was 6.72% in veterinary professionals attending a conference for mixed animal practice, which took place in Czech Republic, confirming the isolation of both animal and nosocomial strains. In this scenario, it appears clear that skin contact with colonized individuals, fomites, or animals may result in either asymptomatic colonization or subsequent clinically significant MRSA disease. Furthermore, globalization, including the increasing of international travel, has facilitated the transmission of multidrug-resistant bacteria such as MRSA across continents, potentially favoring the replacement of existing endemic MRSA with fitter and more transmissible strains [15].

*Staphylococcus pseudintermedius* (*S. pseudintermedius*) is a member of the *Staphylococcus intermedius* group (SIG), together with *Staphylococcus intermedius*, *Staphylococcus delphini*, *Staphylococcus ursi*, and *Staphylococcus cornubiensis* [16]. This coagulase-positive *Staphylococcus* is a normal skin inhabitant and colonizer of healthy dogs [17]. Precisely, *S. pseudintermedius* can be isolated from the nares, oral mucosa, pharynx, forehead, groin, and anus of healthy dogs [18]. Being an opportunistic pathogen, it represents one of the main causes of pyoderma and otitis, along with postoperative wound infections [19,20,21]. In recent years, methicillin-resistant *S. pseudintermedius* strains (MRSP) have also been isolated and identified, and often these MRSP strains showed multidrug-resistant profiles, being also resistant to most of the antimicrobial agents approved for veterinary applications [21,22].

Although *S. pseudintermedius* is not a normal human commensal bacterium, for its zoonotic potential it can be responsible for serious opportunistic infections in humans, particularly affecting pet owners and veterinary personnel [23,24,25,26,27].

According to the International Organization for the Protection of Animals [28] report, in Italy, 13,209,745 dogs and 956,308 cats were registered in the regional registers of pets through 4 February 2022. This number, gradually increased over the years, shows that pet dogs and cats are present in Italian households. In particular, household pet number has risen sharply during the years of the pandemic. The context of the COVID-19 pandemic has provided an opportunity to understand how animals can offer social support to their owners and alleviate heightened symptoms of stress, anxiety, and depression, and contribute to happiness during this major global crisis. In fact, pet dogs and cats may have provided people with a stronger sense of social support, which in turn may have helped reduce some of the negative psychological impacts caused by the COVID-19 pandemic [29]. Contact and interactions between owners and their pets can have benefits [30] but can also facilitate the transmission of zoonotic agents. Indeed, microorganisms transmitted from animals to humans represent about 60% of pathogens, and close contact is essential for transmission [27,31]. However, to estimate the risk of these contacts, more information is needed, such as frequency and intensity.

People can be exposed to zoonoses by direct contact through biting, scratching, sneezing, coughing, licking, or by handling pets and their droppings; or by indirect contact through bedding, food, and water [32]. Furthermore, the possibility of intraspecific transmission should also be considered, for example, during interactions between dogs during playful activities.

The purpose of this study was to identify *Staphylococcus* species isolated from nasal swabs of both healthy and diseased dogs, and those of human origin, obtained from nasal swabs of both owners and veterinary staff. In addition, in order to determine the frequency and intensity of owners’ interactions with their dogs, a survey was conducted among dog-owning households, with the collection of 71 pet-care questionnaires. The antimicrobial resistance profiles of collected strains were also investigated.

## 2. Materials and Methods

### 2.1. Ethical Statement

This study was approved by the Veterinary Service Center of the University of Naples Federico II, Italy (certificate number PG/2021/0009256), in compliance with the Italian Legislative Decree 26/2014, Article 2, implementing Directive 2010/63/EU.

### 2.2. Informed Consent

No animals were used in this study. Samples, represented by only noninvasive nasal swabs, were obtained from dogs being investigated for clinical reasons and for their benefit. Furthermore, nasal swabs were collected from the dogs’ owners and veterinary staff, who agreed to take part in this project by signing an informed consent and performing the nasal swabs by themselves, following the given sampling instructions. After sampling, swabs were held at 4 °C during transport to the bacteriology laboratory.

### 2.3. Participant Groups and Small Pet-Care Questionnaire Survey

In this study, performed in 2022, five different groups of participants were enrolled: I group_ healthy dogs, II group _ healthy dogs’ owners, III group _ diseased dogs, IV group_ diseased dogs’ owners, and V group _ veterinary staff.

**Figure 1:**
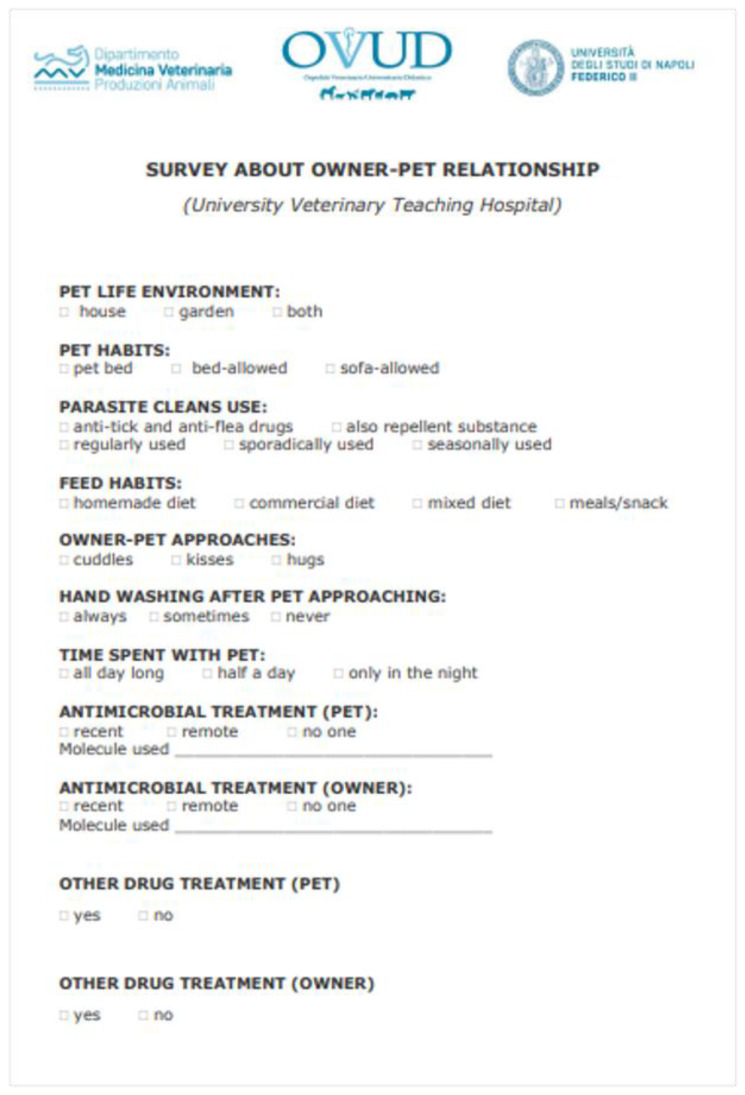
Pet-care questionnaire survey.

Pet owners were asked to complete a questionnaire relating to the care and relationship with their pets (Figure 1). In particular, the questionnaire had the aim to assess some aspects of the dog–owner relationship and to understand if constant and prolonged contact with dogs might represent a potential risk for the transmission of pathogens and their antimicrobial resistance determinants, leading to the selection and sharing of strains with zoonotic potential. The survey consisted of a series of 11 questions having a 2–4 multiple-choice format, and the possibility to provide additional information. The questions focused on the dog–owner relationship, the pet’s lifestyle, and the canine medical history such as recent or previous antimicrobial treatments. This last question was also foreseen for owners. Most patients and people enrolled in this study had not received an antimicrobial treatment for at least two months, classified in the survey as remote antimicrobial therapy (in the last two months/two years), while only few patients received a recent antimicrobial treatment (in the last ten days/one month).

### 2.4. Sampling

Nasal samples were collected from dogs and their owners attending the University Veterinary Teaching Hospital of the Department of Veterinary Medicine and Animal Production (University of Naples “Federico II”, Italy). In particular, the sampling was performed on dogs not affected by skin disorders, referred to the hospital for routine checks or for annual vaccination booster, thereafter, defined healthy, and their owners; but also on dogs clinically suffering from otitis or pyoderma, here indicated as diseased, and their owners. Further nasal swabs were obtained from veterinary staff involved in the daily clinical activity at the University Veterinary Teaching Hospital of Naples.

For each patient, a single swab was inserted about 2/3 cm deep and rotated 360° a couple of times in both nostrils and then placed into Stuart W/O CH (Aptaca Spa, Asti, Italy) transport medium. Within a maximum of 24 h after sampling, specimens, kept at 4 °C, were transferred in an icebox to the bacteriology laboratory of the abovementioned department for bacteriological investigation.

### 2.5. Staphylococcus spp. Isolation and Identification

Once arrived at the laboratory, nasal swabs were streaked on Mannitol salt agar (MSA) plates purchased from Liofilchem Srl (Teramo, Italy). MSA is a selective medium used for isolation, enumeration, and differentiation of *Staphylococcus* spp. After an overnight incubation at 37 °C, the recovered colonies were firstly evaluated for their morphologic and fermentative features (colony morphology, mannitol fermentation) and subjected to rapid standard screening techniques such as Gram’s staining, catalase (Biomérieux, Marcy-l**’**Étoile, France), and oxidase (Liofilchem, Teramo, Italy) reactions. In addition, staphylocoagulase reaction was performed (Oxoid, Ltd., Hampshire, UK).

Thereafter, colonies, grown on MSA, were subcultivated on Columbia CNA agar plates (Liofilchem, Teramo, Italy) and incubated aerobically at 37° for 24 h. Subsequently, the identification of the isolated strains was carried out by using matrix-assisted laser desorption/ionization–time of flight mass spectrometry (MALDI-TOF MS) (Bruker Daltonics Inc., Bremen, Germany), according to manufacturer’s guidelines. Specifically, according to Bruker biotyper’s guidelines, a score of ≥2.0 indicated highly probable species-level identification, a score of 1.70 to 1.99 indicated a secure identification to the genus level, and a score of <1.7 was interpreted as no identification.

*Staphylococcus aureus* ATCC**^®^** 33591^TM^ and *Staphylococcus pseudintermedius* ATCC**^®^** 49444^TM^ were used as quality control strains.

### 2.6. Antimicrobial Susceptibility Testing of Isolated Staphylococci

The susceptibility profiles of the isolated strains were evaluated for 18 antimicrobials, belonging to 11 different classes, by the disk diffusion method on Mueller Hinton agar plates. The tested antimicrobials comprised amoxicillin–clavulanate (AUG, 20/10 µg), ampicillin (AMP, 30 µg), cephalothin (KF, 30 µg), cefoxitin (FOX, 30 µg), ciprofloxacin (CIP, 5 µg), clindamycin (CD, 2 µg), doxycycline (DO, 30 µg), enrofloxacin (ENR, 5 µg), erythromycin (E, 15 µg), gentamicin (CN, 10 µg), levofloxacin (LVX, 5 µg), linezolid (LNZ, 30 µg), nitrofurantoin (F, 300 µg), oxacillin (OX, 1 µg), penicillin (P, 10 IU), sulfamethoxazole–trimethoprim (SXT, 23.75/1.25 µg), tetracycline (TE, 30 µg), and vancomycin (VA, 30 µg), all of which were supplied by Liofilchem Srl (Teramo, Italy). For the classification of the isolated strains as susceptible, intermediate susceptibility, or resistant, the Clinical and Laboratory Standards Institute guidelines [33] were taken into consideration for all tested antimicrobials except for AMP, LVX, and LNZ, for which the European Committee on Antimicrobial Susceptibility Testing guidelines [34] were used.

### 2.7. Statistical Analysis

All diagnostic results generated by the Microbiological Diagnostic Laboratory were recorded and entered into a Microsoft 365 Excel™ spreadsheet for successive analysis. Descriptive statistical analysis was employed to evaluate the prevalence of isolated *Staphylococcus* species and the frequencies of antibiotic resistance among recovered isolates. All graphics were made by using Excel software. The statistical significance level between variable groups was investigated using two-tailored Fisher’s exact test (Social Science Statistics https://www.socscistatistics.com, accessed on 20 May 2023) and *p*-values ≤ 0.05 were considered statistically significant at 95% confidence interval.

## 3. Results

### 3.1. Pet-Care Questionnaire Survey

During this study, 71 questionnaire surveys were collected. In particular, 28 and 43 were filled by healthy dogs’ and diseased dogs’ owners, respectively. The survey consisted of a series of 11 questions having a 2–4 multiple-choice format, with the possibility to provide additional information, above all for the question concerning the use of antimicrobials. In this case, the administered antimicrobial agent was always indicated; for diseased dogs, the reason for presentation in the University Veterinary Teaching Hospital was also requested. In most cases, the diseased dogs were referred to the hospital because of skin disorders, such as otitis externa, pyoderma, hair loss, presence of scales and/or dandruff, or presence of skin wounds.

The answers are summarized in Table 1.

**Figure 2:**
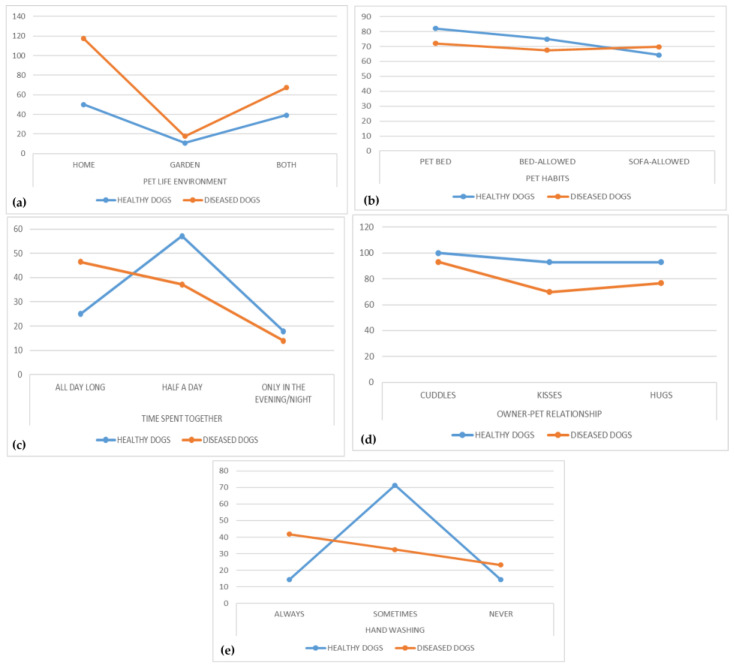
Comparison of answers between healthy and diseased dogs’ owners: (**a**) pet life environment; (**b**) pet habits; (**c**) time spent together; (**d**) owner–pet relationship; (**e**) hand washing.

Comparing the answers obtained, it should be noted that, despite the different numbers in the two sampled owners groups, the behaviors and habits of the pet owners are quite similar (Table 1, Figure 2), especially concerning the intimate pet–owner relationship and the intensity of the contact with high percentages of both healthy and diseased dogs’ owners giving cuddles (100% and 93%, respectively), kisses (93% and 70%, respectively), and hugs (93% and 77%, respectively) to their dogs (Figure 2c,d). Furthermore, 75% of healthy dogs’ owners and 67% of diseased dogs’ owners allowed their pets the access to bed (Figure 2b). In addition, reduced attention given to hand washing was observed among owners, with 14% and 23% of healthy and diseased owners, respectively, never washing their hands after a contact with their dog (Table 1, Figure 2e). However, a significantly higher number (*p* < 0.05) of diseased dogs’ owners (18/43; 42%) indicated always washing their hands after interaction with their pet compared to healthy dogs’ owners (4/28; 14%).

### 3.2. Identification of Staphylococcus spp. from Nasal Swabs of Pet Dogs and Their Owners

During the investigation period, a higher number of samples were processed than reported in this study, as in dogs affected by skin disorders, other specimens, besides nasal swabs, were also collected (e.g., auricular and skin swabs, hair, etc.) to perform a proper diagnosis. Therefore, this study focused only on *Staphylococcus* spp. strains isolated from the nasal swabs of both healthy and diseased dogs and those of their respective owners, in order to evaluate and highlight a possible dog–owner correspondence.

Only 52% dog–owner couples (37/71 dog–owner couples) had positive results for *Staphylococcus* spp. strains during the bacteriological investigation. Staphylococci recovered from canine and human nasal swabs were identified by MALDI-TOF MS with a log(score) ≥ 2.2, indicating a highly probable species-level identification.

In the group of the 13 healthy dogs positive to bacteriological examination, *S. pseudintermedius* was the most frequently isolated strain (46.1%, 6/13 strains), followed by *S. aureus* and *S. epiderdimis*, showing both an isolation frequency of 23% (3/13 strains). A strain of *S. xylosus* was also identified (7.6%).

*S. aureus* and *S. epidermidis* were both the predominant species (38.4%; 5/13 strains) among the staphylococcal strains isolated from the nasal swabs in the group_ healthy dogs’ owners. *S. haemolyticus* was also recovered with an isolation frequency of 23% (3/13 strains).

A comparison of the staphylococcal identifications among the 13 owner–healthy dog couples highlighted a higher occurrence for the following bacterial pairings (Table 2): *S. epidermidis*–*S. pseudintermedius* (23%; 3/13), *S. aureus*–*S. epidermidis* (15.4%; 2/13), *S. haemolyticus*–*S. aureus* (15.4%; 2/13), *S. aureus*–*S. pseudintermedius* (15.4%; 2/13), *S. aureus*–*S. aureus* (7.7%; 1/13), *S. haemolyticus*–*S. pseudintermedius* (7.7%; 1/13), *S. epidermidis*–*S. xylosus* (7.7%; 1/13), and *S. epidermidis*–*S. epidermidis* (7.7%; 1/13).

As for healthy dogs, also in the group composed of diseased dogs, *S. pseudintermedius* was the most commonly identified staphylococcal species (75%; 18/24 strains) from nasal swabs, followed by *S. lugdnensis* (8.3%; 2/24), and *S. hyicus*, *S. lentus*, *S. sciuri, S. simulans*, all four showing an isolation rate of 4.2% (1/24 strains).

Interestingly, *S. epidermidis* was the most predominant microorganism (66.7%; 16/24) in the diseased dogs’ owner group. *S. aureus* and *S. pseudintermedius* were also recovered, but with lower frequencies: 29.2% (7/24 strains) and 4.2% (1/24 strains), respectively.

The comparison of staphylococcal identifications among the 24 owner–diseased dog couples highlighted a higher occurrence for the following bacterial pairings (Table 3): *S. epidermidis*–*S. pseudintermedius* (45.8%; 11/24), *S. aureus*–*S. pseudintermedius* (25%; 6/24), *S. epidermidis*–*S. lugdunensis* (8.3%; 2/24), *S. epidermidis*–*S. sciuri* (4.2%; 1/24), *S. epidermidis*–*S. lentus* (4.2%; 1/24), *S. epidermidis*–*S. hyicus* (4.2%; 1/24), *S. pseudintermedius*–*S. pseudintermedius* (4.2%; 1/24), *S. aureus*–*S. simulans* (4.2%; 1/24).

The analysis of obtained identification highlighted isolation of 12 *S. aureus* from 37 owners (32.4%) and 3 from 37 dogs (8.1%), whereas *S. pseudintermedius* isolation frequencies were 64.8% (24/37) and 2.7% (1/37) for dogs and owners, respectively.

### 3.3. Identification of Staphylococcus spp. from Nasal Swabs of Veterinary Staff

The possible dog–human transmission of *Staphylococcus* spp. was also investigated among the veterinary staff (V group) at the University Veterinary Teaching Hospital of Naples, considering their daily contact with small animal pets referred to the hospital. In particular, 34 nasal swabs were collected, of which 19/34 (55.9%) swabs yielded *Staphylococcus* spp. isolates, while 15/34 (44.1%) nasal swabs did not yield any *Staphylococcus* spp. growth. In contrast to the results obtained for diseased dogs’ owners where *S. epidermidis* was predominant, *S. aureus* (52.6%; 10/19 strains), followed by *S. pseudintermedius* (26.3%; 5/19 strains), *S. epidermidis* (15.8%; 3/19 strains), and *S. haemolyticus* (5.3%; 1/19 strains) were detected in nasal specimens of veterinary staff.

### 3.4. Antimicrobial Resistance Profiles of Staphylococcus spp. Strains Recovered from Dogs’ and Owners’ Nasal Swabs

In the current study, the antimicrobial resistance profiles of *Staphylococcus* spp. strains isolated from both healthy and diseased dogs and their respective owners were evaluated and compared.

**Figure 3:**
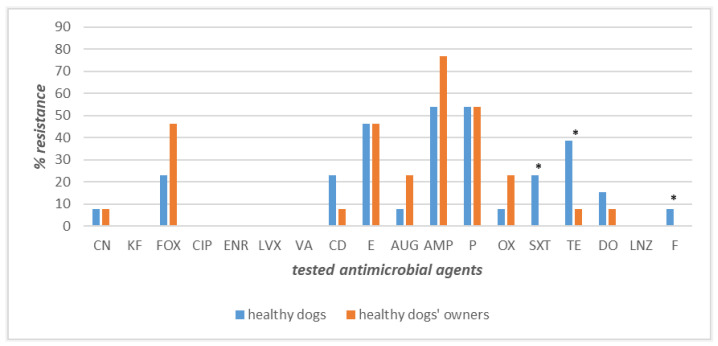
Antimicrobial resistance frequencies of *Staphylococcus* spp. isolated from healthy dogs and their owners. **Legend_** Tested antimicrobials: amoxicillin–clavulanate (AUG), ampicillin (AMP), cephalothin (KF), cefoxitin (FOX), ciprofloxacin (CIP), clindamycin (CD), doxycycline (DO), enrofloxacin (ENR), erythromycin (E), gentamicin (CN), levofloxacin (LVX), linezolid (LNZ), nitrofurantoin (F), oxacillin (OX), penicillin (P), sulfamethoxazole–trimethoprim (SXT), tetracycline (TE), and vancomycin (VA). * *p* < 0.05 for SXT, TE, F.

The strains recovered from the 13 owner–healthy dog couples displayed high levels of susceptibility for most of the antimicrobial agents tested. Precisely, 100% of the canine and human isolates were found to be susceptible to cephalothin, ciprofloxacin, enrofloxacin, levofloxacin, vancomycin, and linezolid (Figure 3). Conversely, the highest levels of resistance were observed for ampicillin (77% owners and 54% dogs), penicillin (54% in both), and erythromycin (43% in both). Different percentages of resistance, with higher values among healthy dogs’ strains than human ones, were observed for tetracycline (39% vs. 8%), clindamycin (23% vs. 7.7%), and doxycycline (15.4% vs. 7.7%), whilst an inverted trend, with higher values among human isolates, was obtained for cefoxitin (42.2% vs. 23%), oxacillin (23% vs. 7.7%), and amoxicillin–clavulanate (23% vs. 7.7%) (Figure 3). Gentamicin was the only antimicrobial to which a lower and identical value of resistance was recorded (7.7%) (Figure 3). The differences in resistance frequencies between canine and human strains were significantly higher (*p* < 0.05) in healthy dogs than their owners for tetracycline, nitrofurantoin, and sulfamethoxazole–trimethoprim (Figure 3).

**Figure 4:**
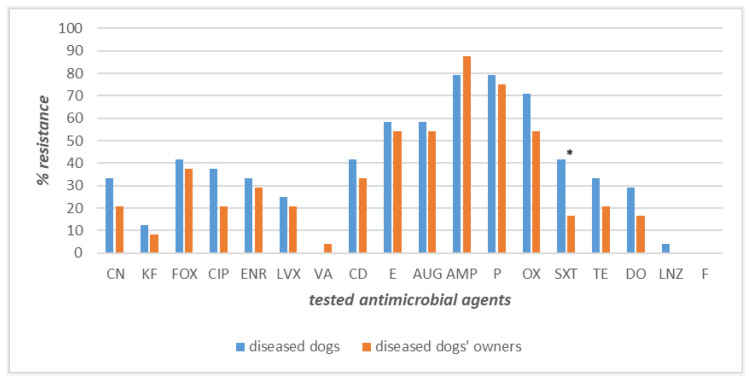
Antimicrobial resistance frequencies of *Staphylococcus* spp. isolated from diseased dogs and their owners. **Legend_** Tested antimicrobials: amoxicillin–clavulanate (AUG), ampicillin (AMP), cephalothin (KF), cefoxitin (FOX), ciprofloxacin (CIP), clindamycin (CD), doxycycline (DO), enrofloxacin (ENR), erythromycin (E), gentamicin (CN), levofloxacin (LVX), linezolid (LNZ), nitrofurantoin (F), oxacillin (OX), penicillin (P), sulfamethoxazole–trimethoprim (SXT), tetracycline (TE), and vancomycin (VA). * *p* < 0.05 for SXT.

Intriguingly, higher resistance levels were recorded for the strains isolated from the 24 diseased dog–owner couples (Figure 4). Specifically, the highest values of resistance were exhibited by ampicillin (87% in owners and 79% in dogs), penicillin (79% in dogs and 75% in owners), oxacillin (71% in dogs, 54% in owners), amoxicillin–clavulanate, and erythromycin, displaying the same level both in diseased dogs and their owners (58% and 54%, respectively). Important differences in resistance frequencies were found for sulfamethoxazole–trimethoprim, ciprofloxacin, and linezolid against which canine strains showed higher percentages of resistance than owner strains: 42%, 38%, and 4%, respectively (Figure 4). The differences in resistance frequencies between canine and human strains were significantly higher (*p* < 0.05) in dogs suffering from bacterial skin disorders than their owners only for sulfamethoxazole–trimethoprim (Figure 4).

**Figure 5:**
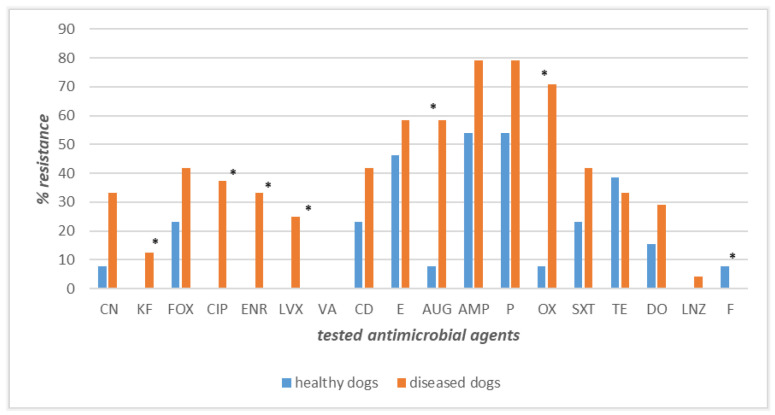
Antimicrobial resistance frequencies of *Staphylococcus* spp. isolated from diseased and healthy dogs. **Legend_** Tested antimicrobials: amoxicillin–clavulanate (AUG), ampicillin (AMP), cephalothin (KF), cefoxitin (FOX), ciprofloxacin (CIP), clindamycin (CD), doxycycline (DO), enrofloxacin (ENR), erythromycin (E), gentamicin (CN), levofloxacin (LVX), linezolid (LNZ), nitrofurantoin (F), oxacillin (OX), penicillin (P), sulfamethoxazole–trimethoprim (SXT), tetracycline (TE), and vancomycin (VA). * *p* < 0.05 for KF, CIP, ENR, LVX, AUG, OX, F.

A comparison of the antimicrobial resistance profiles of staphylococci isolated from healthy and diseased dogs underlined higher resistance values among strains isolated from diseased dogs than those recovered from healthy dogs, except for only tetracycline, where, instead, lower resistance levels were recorded than in healthy dogs (Figure 5). It is worth noting, however, that the highest resistance levels in diseased dogs were shown especially by ampicillin (79%), penicillin (79%), and oxacillin (71%), indicative of a greater spread of methicillin-resistant strains. Furthermore, the staphylococcal strains isolated from diseased dogs were also resistant to ciprofloxacin (38%), enrofloxacin (33%), levofloxacin (25%), cephalothin (13%), and linezolid (4.16%).

The differences in resistance frequencies among canine strains were significantly higher (*p* < 0.05) in diseased dogs than healthy dogs for the following antimicrobials: cephalothin, ciprofloxacin, enrofloxacin, levofloxacin, amoxicillin–clavulanate, oxacillin, and nitrofurantoin (Figure 5).

**Figure 6:**
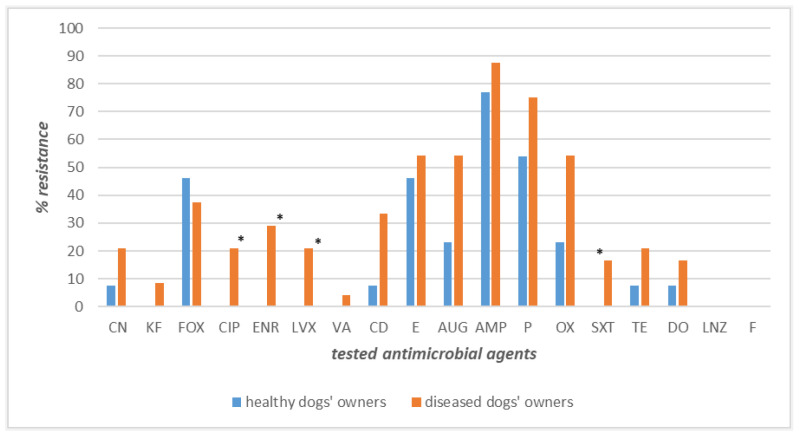
Antimicrobial resistance frequencies of *Staphylococcus* spp. isolated from diseased dogs’ and healthy dogs’ owners. **Legend_**Tested antimicrobials: amoxicillin–clavulanate (AUG), ampicillin (AMP), cephalothin (KF), cefoxitin (FOX), ciprofloxacin (CIP), clindamycin (CD), doxycycline (DO), enrofloxacin (ENR), erythromycin (E), gentamicin (CN), levofloxacin (LVX), linezolid (LNZ), nitrofurantoin (F), oxacillin (OX), penicillin (P), sulfamethoxazole–trimethoprim (SXT), tetracycline (TE), and vancomycin (VA). * *p* < 0.05 for CIP, ENR, LVX, SXT.

In Figure 6, the antimicrobial resistance frequencies recorded for the staphylococci isolated from healthy dogs’ and diseased dogs’ owners are compared. In this context, outstanding resistances were exhibited by ampicillin (88% for diseased dogs’ owners vs. 78% for healthy dogs’ owners), penicillin (75% for diseased dogs’ owners vs. 54% for healthy dogs’ owners), and oxacillin and amoxicillin–clavulanate (54% for diseased dogs’ owners vs. 23% for healthy dogs’ owners, same rates for both molecules). Resistance to enrofloxacin (30%), ciprofloxacin and levofloxacin (21%), vancomycin (4%), and cephalothin (8%) were observed only for the strains isolated from diseased dogs’ owners.

The differences in resistance frequencies between human strains were significantly higher (*p* < 0.05) in diseased dogs’ owners than healthy dogs’ owners for the following antimicrobials: ciprofloxacin, enrofloxacin, levofloxacin, and sulfamethoxazole–trimethoprim (Figure 6).

In addition, the presence of the same bacterial species (*S. aureus*, *S. pseudintermedius*, *S. epidermidis*) with similar antimicrobial resistance profiles in dogs and their owners was detected in only three cases, suggesting potential bacterial transmission. However, further molecular profiling studies are needed to confirm this hypothesis and to possibly demonstrate at least the origin from a same ancestry.

### 3.5. Antimicrobial Resistance Profiles of Staphylococcus spp. Strains Recovered from Nasal Swabs of Veterinary Staff

All the strains collected from the veterinary staff showed 100% of susceptibility to cephalothin, gentamicin, levofloxacin, nitrofurantoin, vancomycin, and linezolid. Differently, high values of resistance were observed against beta-lactams with a resistance rate of 89.4% for ampicillin and penicillin, followed by amoxicillin–clavulanate (78.9%), oxacillin (57.8%), and cefoxitin (52.6%). Among non-beta-lactam antibiotics, high-level resistance to erythromycin (73.6%) and clindamycin (57.8%) was observed. Furthermore, additional resistances to antibiotics belonging to the following classes were observed: (i) fluoroquinolones, with enrofloxacin and ciprofloxacin exhibiting a resistance of 47.3% and 36.8%, respectively; (ii) tetracyclines, with the same resistance to both doxycycline and tetracycline (42.1%); (iii) sulfonamides, with 36.8% of resistance to sulfamethoxazole–trimethoprim. In addition, the lowest-level resistance was displayed by the three strains identified as *S. epidermidis*.

## 4. Discussion

In recent years, the consideration of dogs as potential reservoirs of opportunistic and antimicrobial-resistant pathogens has increased, even though it is not completely clear how the extent and the timing of the transmission of opportunistic pathogens as *Staphylococcus* spp. between household dog and owner occurs [27,35]. During the COVID-19 pandemic, the number of pet dogs has notably increased. In fact, in the European Union there are more than 72 million households that own dogs [36], of which 25% is in Italy [37]. Furthermore, it is worth noting that pet owners often have intensive and close contact with their pet animals, and this is the reason why the European Medicines Agency has already addressed the minimal knowledge about risk factors and transmission routes for transfer of antimicrobial-resistant pathogens with zoonotic potential between pet animals and humans, also pointing out the risk of antimicrobial resistance transfer from pet dogs and cats [38].

In this study, the results of the questionnaire completed by the pet owners underlined a very close relationship with their dogs. Indeed, in most cases (61%), dogs lived at home, and 70% of household dogs had free access to bed, sleeping with owners, and 68% to the sofa. These results agree with those from other studies. In particular, Stull et al. [39] described that in Canada, 26% of pet dogs slept in the bed with the owning family children, and this habit was also reported in Netherlands [40,41] and in UK [42], with dogs sleeping under the blankets at rates of 18% and 14%, respectively. Since sleeping in the same bed represents a very intimate direct contact, it really may constitute a potential risk favoring the transmission of zoonotic and opportunistic pathogens (i.e., *Staphylococcus* spp., *Enterobacterales*, etc.) to their owners through the fur or paw pads, and this risk is higher above all when dogs are affected by skin bacterial disorders. Therefore, it would be desirable for the owners to be adequately informed and educated about this potential risk, to responsibly interact with their dogs.

In this study, 81.7% of dog-owning households described a very tender relationship, characterized by hugs and kisses with their dogs, demonstrating a strong dog–owner interaction. Our results are comparable in extent and frequency to those described in another study [30] in which 85.3% of dogs licked the owner’s hands and 49.3% of owners reported being licked in the face (intense contacts). Moreover, in situations where saliva can be transferred, for example, when a dog licks its owner’s face or hand, microorganisms can be easily transferred from the dog to the owner or *vice versa*. In this study, 30.9% of participants claimed to always wash their hands after petting or touching their dog, a higher rate compared to that found in another report, where only 15% of respondents washed their hands [40]; 19.7% of the pet owners involved in our study stated that they never washed their hands. Specifically, our pet-care questionnaire highlighted a significantly greater (*p* < 0.05) attention given to hand washing by diseased dogs’ owners, with 42% of them always washing their hands, than healthy dogs’ owners, with only 14% declaring to always wash hands. This result is probably linked to the presence of skin disorders in their dogs at the time of the interview. Surprisingly, despite the attention given to hand washing; diseased dogs’ owners referred to occasional use of anti-tick and anti-flea drugs and repellents.

The oral cavity and the skin of dogs host a varied microbial flora, including many species of opportunistic pathogens, which can be responsible for even serious health problems in high-risk individuals (young, elderly, pregnant women, and immunocompromised people). Transmission generally occurs through the bite, contact with saliva, mucous membranes, or on an open wound; this can cause serious infections in high-risk patients [43,44]. Indeed, it is known that hand washing is a critical point, a moment to pay attention to, as it is one of the best hygiene measures to remove germs, avoid getting sick, and prevent the spread and the transmission of opportunistic germs to others.

In this study, from a total of 172 nasal samples of both canine and human origin, 60.5% were positive for *Staphylococcus* spp. Among identified canine nasal staphylococcal strains, *S. pseudintermedius* was the most predominant identified bacterium (46%). This high *S. pseudintermedius* occurrence is not surprising, as other published studies described *S. pseudintermedius* as a normal constituent of the microbial flora in the nasal cavities of dogs [45], but also as the main opportunistic canine pathogen associated with canine skin disorders, such as otitis externa and pyoderma [21,46,47,48,49]. Moreover, this opportunistic microorganism has been reported as responsible for diseases in humans, associated with contact with dogs, suggesting zoonotic transmission [16,50,51]. Transmission can be hypothesized for the dog–owner interaction in our study, where both the dog suffering from pyoderma and its owner were positive for *S. pseudintermedius* strains, showing a comparable antimicrobial resistance profile. Even though humans are known not to be its natural host, they can be transiently colonized by *S. pseudintermedius*, including methicillin-resistant *S. pseudintermedius* (MRSP). In this regard, Guardabassi et al. [52] demonstrated that owners of dogs suffering from pyoderma were more likely to be positive for *S. pseudintermedius* compared to people not having daily contact with dogs, but there was no evidence of colonization at the time of a second sampling 2 months later, suggesting that *S. pseudintermedius* long-term colonization is uncommon in humans. This may be one of the reasons why currently the prevalence of human colonization is still unknown; the other one could be represented by the misidentification, above all in past years, of this pathogen as *S. aureus*, due to the lack of knowledge of this bacterial species in human medicine [53,54].

In two other cases, it was possible to recover the same bacterial strain both in the dog and its owner. Precisely, in one case, a strain of *S. epidermidis* was isolated from the nasal swab of the dog and its owner; in the other case, the isolated strain was represented by *S. aureus*. In these two cases, the strains of different origins showed overlapping phenotypes and similar antimicrobial resistance profiles, and led us to hypothesize a possible transmission from the owner to dog, as these microorganisms are more commonly isolated in humans [55,56]; however, this hypothesis also needs support from molecular-typing studies.

Concerning the species belonging to the genus *Staphylococcus* isolated from the nasal cavities of dogs’ owners, *S. aureus* was the most commonly isolated species, as well as from the nasal cavities of veterinary staff. These results agree with those of other studies, since *S. aureus* is a normal constituent of the microbial flora of both skin and nasal mucous membranes of humans, found in up to 68% of examined cases [57,58]. However, it has also been shown how the skin microbiome is primarily dependent on the physiology of the skin site [59] and can be greatly influenced by a series of factors such as age, environment rather than the habits of the person (smokers and nonsmokers), and the onset of pathologies [60].

In this study, another *Staphylococcus* species often isolated from human nasal specimens was *S. epidermidis*, a coagulase-negative *Staphylococcus* (CoNS) described as the species that competes with *S. aureus* for stable colonization in human nasal mucosa. Furthermore, it is also able to produce a serine protease (Esp), which inhibits *S. aureus* biofilm formation [61], an activity documented in many studies carried out in hospitalized infants, adolescents, and adults [61,62].

*Staphylococcus aureus* (29.4%) followed by *S. pseudintermedius* (14.7%) were the most frequently isolated strains from the culture of nasal swabs sampled among the staff working at the University Veterinary Teaching Hospital. These results partially agree with those obtained for pet owners’ nasal samples, with *S. aureus* and *S. epidermidis* as the most prevalent ones. This variation and replacement of *S. epidermidis* with *S. pseudintermedius* could be related to the continuous contact that the veterinary staff has daily with diseased dogs and cats, especially those affected by skin infections, such as pyoderma and otitis. Although traditionally *S. pseudintermedius* is not considered a risk for humans, an increase in its zoonotic transmission has been reported above all for MRSP in recent years [24,25,26]. In this context, the veterinary environments (hospital, clinics) seem to play an important role in the dissemination of MRSP between animals and people, especially people who have constant contact with pets (veterinary personnel and pet owners) [20,63]. Paul et al. [63] pointed out that 3.9% of small animal dermatologists attending a national veterinary conference in Italy carried MRSP in their nasal cavities. This is an important rate of MRSP carriage, considering the relative rare occurrence in humans and, consequently, it might be considered an occupational risk [63].

Referring to the antimicrobial resistance profiles of isolated staphylococci, high levels of resistance to penicillins (oxacillin and penicillin) were recorded. In particular, the highest level of resistance, statistically significant, against oxacillin was observed for the strains isolated from diseased dogs (71%) compared to the healthy dogs (7.7%), representing an alarming result, since MRSP growing spread has become a relevant issue, causing great concern in veterinary medicine [64,65,66]. The emergence of MRSP has for some time highlighted the need for careful long-term surveillance of this strain. However, the high rate of resistance (46% for healthy dog–owner couples and >50% for diseased dog–owner couples) to erythromycin, a macrolide widely used for the treatment of human and animal infections but not generally used in canine infections, was here exhibited by staphylococci isolates of both human and canine origin. The increased spread of bacteria showing worrying antimicrobial resistance profiles underlines the crucial role of the One Health Approach in helping to better control the antimicrobial resistance issue and, consequently, the importance of prudent use and proper administration of antimicrobials in both pet animals and humans [67,68].

In conclusion, the results of this study suggested the potential risk of transmission of *S. aureus* from dog owners to their dogs and of *S. pseudintermedius* from dogs to their owners and to veterinary staff. Continuous monitoring is desirable to define the circulation of antimicrobial-resistant staphylococci and to further explore the possibility of animal–human–animal transmission through epidemiological studies concerning interspecies transmission. Particular attention is required for strains such as *S. pseudintermedius* and *S. aureus*, which are important candidates for “One Health” initiatives.

## 5. Conclusions

Dog–human relationships, which have become closer and more continuous over time, can bring benefits but can also allow the possible transmission of opportunistic pathogens. The high prevalence of *S. pseudintermedius* among dogs, *S. aureus* and *S. epidermidis* among owners, and *S. aureus* and *S. pseudintermedius* among veterinary staff suggests the possible transmission of these staphylococci between people and dogs or *vice versa*. In any case, this hypothesis should be confirmed by molecular analysis techniques such as whole genome sequencing. In addition, monitoring of antimicrobial resistance profiles is desirable to evaluate the emergence of new resistances among the circulating strains, along with their transmissibility and pathogenicity. Thus, adequate prevention and control measures for domestic infections control could be better applied.

## Figures and Tables

**Table 1:** Pet-owner interview questions and answers.

		Healthy (N.28)	Diseased (N.43)
**Pet life environment**	Home	14 (50%)	29 (67%)
	Garden	3 (11%)	3 (7%)
	Both	11 (39%)	11 (26%)
**Pet habits**	Pet bed	23 (82%)	31 (72%)
	Bed-allowed	21 (75%)	29 (67%)
	Sofa-allowed	18 (64%)	30 (70%)
**Use of parasite cleaners**	Anti-tick and -flea drugs	26 (93%)	36 (84%)
	Repellent substances	10 (36%)	7 (16%)
	Regularly used	15 (54%)	15 (35%)
	Sporadically used	5 (18%)	5 (12%)
	Once a month	9 (32%)	6 (14%)
	Seasonally used	7 (25%)	13 (30%)
**Feed habits**	Homemade diet	2 (7%)	4 (9%)
	Commercial diet	14 (50%)	30 (70%)
	Mixed diet	12 (43%)	9 (21%)
	Snacks	18 (64%)	6 (14%)
**Owner–pet** **relationship**	Cuddles	28 (100%)	40 (93%)
	Kisses	26 (93%)	30 (70%)
	Hugs	26 (93%)	33 (77%)
**Hand washing**	Always	4 (14%)	18 (42%)
	Sometimes	20 (71%)	15 (35%)
	Never	4 (14%)	10 (23%)
**Time spent together**	All day long	7 (25%)	20 (47%)
	Half a day	16 (57%)	16 (37%)
	Only in the evening/night	5 (18%)	7 (16%)
**Drug treatment** **(dog)**	Recent	4 (14%)	6 (14%)
	Remote	6 (21%)	20 (47%)
	No one	18 (64%)	17 (40%)
**Antibiotic** **administration** **(dog)**	Yes	6 (21%)	6 (14%)
	No	22 (79%)	37 (86%)
**Drug treatment** **(owner)**	Recent	0	0
	Remote	19 (68%)	10 (23%)
	No one	9 (32%)	33 (77%)
**Antibiotic** **administration** **(owner)**	Yes	5 (18%)	6 (14%)
	No	23 (82%)	37 (86%)

**Table 2:** Occurrence of *Staphylococcus* spp. identified in owner–healthy dogs couples.

Strains from Owners	Strains from Healthy Dogs	Occurrence (%)
*S. epidermidis*	*S. pseudintermedius*	23%
*S. aureus*	*S. epidermidis*	15.4%
*S. haemolyticus*	*S. aureus*	15.4%
*S. aureus*	*S. pseudintermedius*	15.4%
***S. aureus***	***S. aureus***	**7.7%**
*S. haemolyticus*	*S. pseudintermedius*	7.7%
*S. epidermidis*	*S. xylosus*	7.7%
***S. epidermidis***	***S. epidermidis***	**7.7%**

**Table 3:** Occurrence of *Staphylococcus* spp. identified in owner–diseased dogs couples.

Strains from Owners	Strains from Diseased Dogs	Occurrence (%)
*S. epidermidis*	*S. pseudintermedius*	45.8%
*S. aureus*	*S. pseudintermedius*	25%
*S. epidermidis*	*S. lugdunensis*	8.3%
*S. epidermidis*	*S. sciuri*	4.2%
*S. epidermidis*	*S. lentus*	4.2%
*S. epidermidis*	*S. hyicus*	4.2%
***S. pseudintermedius***	***S. pseudintermedius***	**4.2%**
*S. aureus*	*S. simulans*	4.2%

## Data Availability

Not applicable.
